# Impact of Successful Weight Loss Maintenance on Serum Lipids and Glucose Concentrations of Previous Participants of a Weight Loss Programme in Accra, Ghana

**DOI:** 10.1155/2019/4729040

**Published:** 2019-04-01

**Authors:** Sandra Ayisi Addo, Christiana Nti, Frederick Vuvor, Jonathan Adjimani, Matilda Steiner-Asiedu

**Affiliations:** ^1^Department of Nutrition and Food Science, University of Ghana, Legon-Accra, Ghana; ^2^Department of Family and Consumer Sciences, University of Ghana, Legon-Accra, Ghana; ^3^Department of Biochemistry, Cell and Molecular Biology, University of Ghana, Legon-Accra, Ghana

## Abstract

**Background and Aim:**

There is a need to investigate the long-term impact of successful weight loss maintenance on blood lipids and glucose concentrations in populations within Africa, where obesity and cardiovascular disease (CVD) rates are increasingly becoming a public health threat. The aim of this study was to compare the serum lipid and glucose concentrations of successful and unsuccessful weight loss maintainers who previously participated in the Nutriline Weight Loss Programme (NWLP) in Accra, Ghana.

**Methods:**

112 participants were randomly selected to participate in this cross-sectional study. Baseline and end of weight loss programme anthropometric and programmatic data were accessed via the NWLP archival database. On follow-up, anthropometric data, physical activity, dietary behaviour, serum lipid, and glucose indices were taken. Successful weight loss maintainers (SWLM) were defined as those achieving at least 5% weight loss below the baseline weight at follow-up, otherwise unsuccessful (UWLM).

**Results:**

The adjusted serum total cholesterol (TC) concentration was significantly lower for SWLM (5.17 ± 0.99 mmol/L) compared to UWLM (5.59 ± 1.06 mmol/L). Serum low-density lipoprotein (LDL), high-density lipoprotein (HDL), fasting blood glucose (FBG), and glycosylated haemoglobin (HbA1c) concentrations for SWLM versus UWLM did not differ significantly and were as follows: 3.58 ± 0.92 mmol/L versus 3.87 ± 0.99 mmol/L, 1.22 ± 0.38 mmol/L versus 1.17 ± 0.32 mmol/L, 4.48 ± 0.72 mmol/L versus 4.73 ± 1.00 mmol/L, and 5.52 ± 0.39% versus 5.59 ± 0.59%, respectively. Triglyceride (TG) concentration was significantly (*P* < 0.001) lower for SWLM (0.79 ± 0.28 mmol/L) compared to UWLM (1.17 ± 0.51 mmol/L). After adjusting for covariates, it was no longer significant. Additionally, there was no significant association between weight loss maintenance success and having a normal status for selected lipids and glucose parameters.

**Conclusion:**

SWLM had a significantly lower serum TC compared to UWLM. In addition, a greater proportion of SWLM had normal values for TC, TG, HbA1c, and LDL out of the six parameters measured although not statistically significant.

## 1. Introduction

Cardiovascular diseases (CVDs) refer to diseases of the heart and blood vessels and are the leading cause of mortality worldwide, responsible for 17.9 million deaths yearly which represents a third of all global deaths [1]. More than 75% of CVD deaths occur in low- and middle-income countries [1]. In Sub-Saharan Africa, 9.2% of deaths are attributable to CVD which is the leading cause of death in adults over 45 years of age [2]. In Ghana, CVDs were the leading cause of institutional mortality and accounted for 14.5% of total deaths in 2008 [3]. The World Health Organization estimated the probability of dying from four major noncommunicable diseases (CVD, cancer, diabetes, and chronic respiratory disease) in Ghana for persons within the age of 30–70 years to be 20% [4]. Cardiovascular diseases such as stroke and coronary heart disease ranked first and second, respectively, in the top 50 causes of death in Ghana in 2018 [5].

Studies indicate that excess body weight is linked to the development of conditions such as dyslipidaemia and elevated blood glucose concentration (type 2 diabetes) which increase the risk for CVDs [6–10]. Obesity prevalence is speedily increasing in Africa and other developing countries compared to the developed world where the rate of increase seems to have generally slowed down [11–13]. In Ghana, past demographic health surveys (1993–2014) have shown consistent increases in the proportion of overweight/obese women (15–49 years) ranging from 13% to 40% [14–17]. The current prevalence of obesity in Ghanaian adults is estimated at 43% [18].

Intentional weight loss of at least 5% below baseline weight results in favourable changes in various health parameters such as serum lipids and glucose concentrations, contributing to a reduction in the risk for CVDs [19–21]. The evidence supporting the health benefits of intentional weight loss is however often limited to studies investigating the benefits just before or at the end of the weight loss intervention [19, 22–25]. Additionally, most of the studies investigating the sustained health benefits derived from intentional weight loss are usually short-term studies of up to two years post weight loss intervention [26–29]. The “Look AHEAD” study is one of the few studies that investigated the sustained health benefits of lifestyle intervention over a longer period (4 years), and this was done in a diabetic population [30]. Diabetics constitute a group that may have elevated baseline concentrations of biochemical parameters such as serum glucose and lipids, thus enhancing the likelihood of greater improvements in these parameters following intentional weight loss [19, 21]. Furthermore, most of the studies investigating either the immediate or long-term effects of weight loss on CVD risk have done so in populations living in the developed world. There is therefore the need to investigate the long-term impact of successful weight loss maintenance on blood lipids and blood glucose concentrations in populations within Africa, where obesity and CVD rates are increasingly becoming a public health threat.

## 2. Materials and Methods

The aim of this study was to compare the serum lipid and glucose concentrations of nondiabetic successful and unsuccessful weight loss maintainers who previously participated in a weight loss programme in Accra, Ghana.

### 2.1. Study Design

A cross-sectional design was used to determine anthropometric data, physical activity, dietary behaviour. serum lipids, and glucose indices at follow-up. Baseline and end of programme anthropometric and programmatic data were accessed via the NWLP archival database.

### 2.2. Nutriline Weight Loss Programme (NWLP)

NWLP is a fee-paying programme run by nutrition professionals. It uses structured and personalised diet plans that supply 1000–1900 calories per day, depending on individual energy needs. Diet plans are combined with behavioural therapy to achieve weight loss targets ranging from 0.5 kg to 1 kg per week. Participants are encouraged to engage in regular physical activity and are required to visit the weight loss centre once every week for a face-to-face encounter with the nutrition professional on duty. During the weekly visits, barriers to weight loss and coping strategies are discussed. At the time of enrolment, the participant chooses a particular programme duration (ranging from 2 to 6 months) based on professional recommendation and cost considerations and is entitled to programme renewal until weight loss goal is achieved.

### 2.3. Study Participants

Study participants completed their first bout of NWLP between the years 2008 and 2016. Out of 550 randomly selected previous NWLP members, 230 members agreed to participate in a larger study on the prevalence of weight loss maintenance success (details and results of the larger study was excluded from this report). We further randomly sub-sampled 112 participants out of the 230 participants for biochemical tests. The randomisation scheme was based on stratification by the year of enrolment as this had a direct bearing on post-treatment time, which had been identified as an independent predictor of weight maintenance success [31]. The sub-sample size used was based on calculations using the standard deviation for a serum total cholesterol of 1.26 mmol/L [32], a margin of error of 5% of the mean for total cholesterol concentration (4.7 mmol/L), as determined by Amoah [32], at a 5% significance level (∝), and a two-tailed critical value (*Z*_∝/2_) of 1.96. Participants who had BMI ≥ 25 at the time of enrolment were able to walk for exercise (self-reported) and were medically fit (self-reported). Participants were excluded from the study if they were below 18 years of age at the time of first enrolment into NWLP, resided outside Accra or Ghana at the time of follow-up, had not completed the NWLP first bout of weight loss at the time of follow-up, had a time lapse from completion of first bout weight loss to the follow-up period that was less than six months, had no review visits to the NWLP centre after day one of the first bout enrolment into the programme, got pregnant at any time point during or after the first bout weight loss with “NWLP”, and had any of the following conditions: diabetes, thyroid disease, cancers, HIV, psychiatric illness, anorexia/bulimia, major organ disease(s), or any other disease that was capable of causing unintentional changes in body weight during or after the initial weight loss programme.

### 2.4. Demographic Information, Anthropometric Data, and NWLP Programmatic Measures

While in NWLP, members had their baseline demographic information, weight and height measurements taken on the first day of enrolment into the programme. Subsequent weekly weights were recorded until the end of programme. Dates of enrolment and of each subsequent visit were recorded for each member. Inputs of these data were made in an electronic database from which computations such as baseline body mass index (BMI), percent weight loss at the end of the weight loss programme, length of stay on the programme, percent of visits made, post-weight loss treatment time, and percent weight regain from the end of programme weight were extracted for consenting participants of this current study. Weight, height, and BMI measurements of consenting participants were also taken at follow-up time, and the percent weight loss at the follow-up period was calculated.

A calibrated Camry digital weighing scale (Camry Electronic Limited, 4 Kang Le Road S., Zhaoqing, Guangdong, China, Model EF954, ISO 9001 certified) was used for weight measurements. Standing height was measured using a stadiometer (Health O Meter, 11800 South Austin Avenue Unit B, Alsip, IL 60803, United States of America). Weights were measured in kilograms to the nearest 0.1 kg and height in centimetres to the nearest 0.1 cm. BMI was derived from the following formula: weight in kilograms divided by the square of height in metres. Participants were weighed in their usual clothing and had no shoes on. The participants with heavy clothes replaced them with light skirts given by the centre. All heavy objects in the pockets of participants were taken out prior to weighing.

### 2.5. Blood Biochemical Measures

This was done at the follow-up period. Venous blood samples were drawn by a certified phlebotomist after a 12–14 hour fast and analysed at an accredited laboratory in Accra, Ghana, using standard laboratory procedures. Selectra Pro S automated chemistry analyser (manufactured by Elitech Clinical Systems SAS-Zone industrielle-61500 SEES France) was used in determining the total cholesterol (TC), high-density lipoprotein (HDL), and triglycerides (TG) concentrations [33, 34]. Low-density lipoprotein (LDL) concentration was calculated from that of the total cholesterol, HDL, and TG, as shown in the following equation:

(1) $$\begin{array}{c} \text{LDL}=\text{total}\;\;\text{cholesterol}-\left(\frac{\text{TG}}{2.2}+\text{HDL}\right). \end{array}$$

Fasting blood glucose (FBG) concentrations of participants were determined using the Accu-Chek performa glucometer and its test strips (manufactured by Roche Diabetes Care Incorporated, 9115 Hague Rd., Indianapolis, IN 46256, United States of America) [35]. The Clover A1c Self system (Infopia Co. Ltd., 132, Anyangcheondong-ro, Dongan-gu, Anyang-si, Gyeonggi-do, 14040, Korea) was used to determine the percentage of glycosylated haemoglobin (HbA1c) in whole blood samples of participants via the boronate affinity assay [36]. Normal blood lipids and glucose values were defined as follows: TC, <5.2 mmol/L; LDL, ≤2.59 mmol/L; HDL, ≥1.03 mmol/L; TG, <1.7 mmol/L; FBG, 3.6–6.4 mmol/L; HbA1c, <6.0%.

### 2.6. Post-Weight Loss Physical Activity and Dietary Behaviour Measures

This was done at the follow-up period. Using an interviewer-administered semi-structured questionnaire, participants were asked to estimate the number of days in a typical week of the past month that they accumulated at least 30 minutes moderate activity or 15 minutes vigorous activity. The World Health Organization's definition and examples of moderate and vigorous activity [37] were read to participants. Participants identified the type, duration, and number of days per week for each activity engaged in. These were further analysed into the respective total minutes of moderate-or-vigorous activity per week and the total moderate-to-vigorous physical activity [38]. Total moderate to vigorous activity duration/week was calculated by adding minutes per week for moderate and vigorous activities. Total moderate-to-vigorous activity was further categorised as none, 1–149 minutes, 150–419 minutes, and ≥ 420 minutes.

Participants were asked if they were practising certain diet-based behavioural strategies: limiting or avoiding fats and oils, limiting food portions at meal time, limiting or avoiding out-of-home eating, and having at least five servings of fruits and vegetables per day (a portion of fruit was defined to be half a cup, and sample cup (240 ml volume) was shown to the participant during the interview). Responses were coded as yes or no, depending on whether the participant endorsed the behaviour or not. To objectively assess whether a behaviour was being limited or avoided, participants were asked a probing question on how often that behaviour was practised and had the following options to choose from ≥once per day/2–6 times per week/once per week/less than once per week/never. Limiting a behaviour was defined as engaging in that behaviour less than once per week, and avoiding a behaviour was defined as never engaging in that behaviour. Fats or oil limiting or avoidance behaviour was assessed based on how often selected high fat/oily foods were eaten. These selected high fat/oil foods were those that were listed as foods to be limited or avoided when participants were on the weight loss programme. Limiting of food portions at meal times was based on participant's self-assessment of whether food portions at meal times were controlled.

### 2.7. Definitions of Outcome Measures

#### 2.7.1. Change in Body Mass Index (BMI) from Baseline to Survey Time

This was calculated by subtracting BMI at survey time from BMI at baseline. A negative BMI change indicated a decreased BMI at survey time, while a positive BMI change indicated an increased BMI at survey time in reference to baseline BMI.

#### 2.7.2. Post-Weight Loss Treatment Time

This is the time lapse (in months) from the date of participant's last review visit during the weight loss intervention to the date the survey was conducted for each participant.

#### 2.7.3. Percent Weight Loss at the End of Weight Loss Programme

This was calculated by dividing weight loss achieved at the end of the weight loss programme by the baseline weight and multiplying by 100. A negative value indicated weight loss occurred at the end of the weight loss programme, while a positive value meant weight gain at the end of the weight loss programme.

#### 2.7.4. Percent Weight Loss at Follow-Up Time

This was calculated by dividing weight loss achieved at the survey time by the baseline weight and multiplying by 100. A negative value indicated weight loss occurred at follow-up, while a positive value meant weight gain at follow-up. Weight loss at follow-up was calculated using baseline weight as the reference.

#### 2.7.5. Percent Weight Regained

This was calculated by subtracting weight at the follow-up time from the weight at the end of the weight loss programme. The resultant figure was divided by the weight at the end of the weight loss programme and multiplied by 100. A negative figure meant the weight loss occurred within this period, while a positive figure implied weight was regained.

### 2.8. Data Analyses

Statistical analyses were conducted using IBM SPSS Statistics for Windows, version 20, Armonk, NY: IBM Corp. Normality of data was assessed using histograms and Shapiro–Wilk's test, where *P* > 0.05 confirmed the normality of data. Descriptive statistics (mean (SD)) were used to characterise continuous variables that were normally distributed, while the median and interquartile range (IQR) were used for non-parametric data. Frequencies and percentages were used to characterise variables that were categorical in nature. Successful weight loss maintainers (SWLM) were defined as those achieving at least 5% weight loss below the baseline weight at the follow-up period. Unsuccessful weight loss maintainers (UWLM) were defined as those achieving less than 5% weight loss below the baseline weight at the follow-up period (this included those who had regressed to the baseline weight or had further regained beyond the baseline weight at the time of follow-up).

Comparisons of SWLM and UWLM were conducted using analysis of covariance (ANCOVA) for normally distributed continuous variables, Mann–Whitney *U*-test for non-parametric continuous variables, and Pearson's chi-square analyses for categorical variables. Fisher's exact test was used in place of Pearson's chi-square test where the participant-expected number in a particular sub-group analysis was less than five. Covariates used in the ANCOVA models were total percent weight loss at the end of weight loss treatment (continuous variable), post-weight loss treatment time (in months) (continuous variable), percent weight regain with reference to end of programme weight (continuous variable), presence of hypercholesterolemia (categorical variable), intake of lipid-lowering medication (categorical variable), gender (categorical variable), age (continuous variable), and follow-up BMI (continuous variable) [19, 26, 31, 39].

## 3. Results

Majority of participants were female (85.7%) and had had a tertiary education (90.2%). More than half (73.2%) were married. The mean baseline age, weight, height, and body mass index (BMI) of participants were 39.7 ± 9.0 years, 95.2 ± 14.9 kg, 1.64 ± 0.07 m, and 35.4 ± 5.1 kg/m^2^, respectively. The mean post-treatment time was 55.0 ± 32.9 months (roughly 4.5 years and ranged from 6 to 110 months) for the entire cohort. SWLM and UWLM did not differ significantly in baseline weight, height, and BMI. Similarly, SWLM and UWLM did not differ in gender status, baseline marital status, and educational status. SWLM were however 3.6 years older in age (*P*=0.036) compared to UWLM (Table 1).

On follow-up of SWLM and UWLM, each had BMI (31.6 ± 4.5 kg/m^2^ versus 36.2 ± 6.0 kg/m^2^, respectively) in the obese range and these were not significantly different from each other. SWLM, however, had a significantly greater reduction in BMI from the baseline to follow-up compared to UWLM who increased in BMI within the same period (−4.9 ± 2.4 kg/m^2^ versus 1.1 ± 1.9 kg/m^2^, respectively, *P* < 0.001) (Table 2). SWLM experienced a significantly higher percentage weight loss at the end of the weight loss programme compared to UWLM (−14.4 ± 6.9% versus −5.5 ± 4.4%, respectively, *P* < 0.001). At the survey time, SWLM achieved a mean percentage weight loss of −13.3 ± 6.2%, while UWLM had a mean percentage weight gain of 3.0 ± 5.0% (*P* < 0.001), with reference to the baseline weight. The median percentage weight regain for UWLM was 7.9% (*P* < 0.001) higher than that of SWLM. The median minutes of physical activity accumulated per week for SWLM and UWLM were each below what was regarded as sufficient (Table 2).

**Figure 1:**
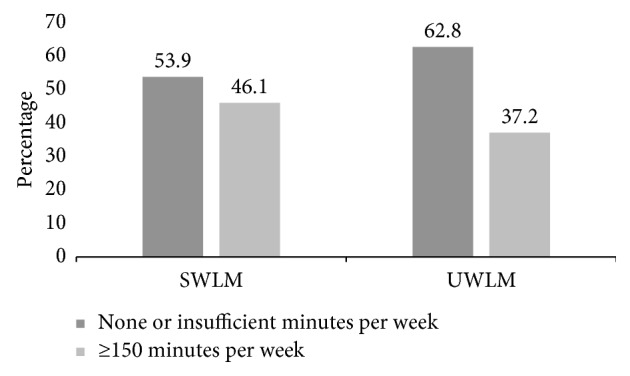
Proportion of SWLM and UWLM engaging in moderate-to-vigorous physical activity (observation was not statistically significant).

A greater proportion of both SWLM and UWLM was neither participating in nor accumulating sufficient minutes per week of moderate-to-vigorous intensity physical activity (Figure 1).

The adjusted mean concentration of TC was significantly lower for SWLM compared to UWLM (5.17 ± 0.99 mmol/L versus 5.59 ± 1.06 mmol/L, respectively, *P*=0.043). This happened only when intake of lipid-lowering medication was added to the list of covariates. We observed that 10% of UWLM versus 3% of SWLM making a total of 13% of the entire cohort used lipid-lowering medication at follow-up. The mean concentrations of LDL, HDL, FBG, and HbA1c did not differ significantly between SWLM versus UWLM in both the adjusted and unadjusted models and were as follows: 3.58 ± 0.92 mmol/L versus 3.87 ± 0.99 mmol/L, 1.22 ± 0.38 mmol/L versus 1.17 ± 0.32 mmol/L, 4.48 ± 0.72 mmol/L versus 4.73 ± 1.00 mmol/L, and 5.52 ± 0.39% versus 5.59 ± 0.59%, respectively. TG concentration was significantly (*P* < 0.001) lower for SWLM (0.79 ± 0.28 mmol/L) compared to UWLM (1.17 ± 0.51 mmol/L) in the unadjusted model (Model 1) and no longer significant after adjusting for covariates in Model 2 (Table 3).

Multiple linear regression analysis of the covariates on triglyceride concentration revealed that the total percent weight loss at the end of the programme, post-treatment time, gender, and follow-up BMI were responsible for the differences in triglyceride concentration for SWLM and UWLM observed in the unadjusted model. Every unit reduction in percent weight loss and every unit increase in post-treatment time and follow-up BMI were responsible for 0.19, 0.20, and 0.31 unit increases, respectively, in triglyceride concentration. On the contrary, every replacement of a male with a female caused a 0.27 unit reduction in triglyceride concentration (Table 4).

There was no significant association between weight loss maintenance success and having a normal status for selected lipid and glucose parameters. There was however a trend of having a higher proportion of SWLM compared to UWLM with normal values for four (TC, TG, HbA1c, and LDL) out of the six parameters assessed (Table 5).

Table 6 shows the prevalence of selected dietary behaviours practised after the weight loss programme. A significantly higher proportion of SWLM limited food portions compared to UWLM. Majority of the entire cohort were neither limiting fats and oil intake in meals nor limiting out-of-home eating. More than half of the cohort was not having the recommended fruit and vegetable servings per day.

## 4. Discussion

The objective of the study was to compare the serum lipids and glucose concentrations for successful and unsuccessful weight loss maintainers. Total cholesterol was slightly but significantly lower in SWLM compared to UWLM after adjusting for lipid-lowering medication usage. This suggests that prior to making this adjustment, the greater use of lipid-lowering medication in UWLM may have led to the lack of significant difference in total cholesterol concentration between SWLM and UWLM. The use of medications (statins) to control lipids was reported to be a potential confounder that significantly modified the association between weight loss and changes in CVD risk factors in the “Look AHEAD” studies [30, 40]. Statins are known to inhibit the activity of HMG CoA reductase and thereby reduce hepatic cholesterol synthesis and upregulate the expression of LDL receptors in the liver. LDL receptors in turn increase the clearance of plasma LDL and thus decrease the concentration of plasma cholesterol [41, 42].

No significant differences were however observed with the concentrations of LDL, HDL, FBG, and HbA1c for SWLM versus UWLM in both the adjusted and unadjusted models. Secondary analysis of female stratified data followed a similar trend of no significant difference in mean concentrations of lipids and glucose for SWLM and UWLM (results not shown). Additionally, secondary analysis using binary logistic regression for the entire cohort data did not reveal any significant associations between the blood parameters and successful weight loss maintenance (results not shown). These findings together could be partly explained by the fact that the entire cohort used for the study was heterogeneous in nature (and not an exclusively high-risk group at risk for conditions such as diabetes, metabolic syndrome which have the likelihood of elevating blood lipids and/or glucose concentrations). Such a heterogeneous group may have had nonelevated mean baseline serum lipid and glucose values, and therefore, the likely improvements in mean serum lipids and glucose concentrations of SWLM may not have been significant to bring about a significant difference between the two groups (SWLM versus UWLM). In a study by Brown and colleagues, greater improvements were seen in a select group of high-risk participants who had higher baseline values of cardiometabolic risk factors compared to the general heterogeneous group [21]. Wadden et al. and colleagues [43] also demonstrated that sustained weight loss of ≥5% below baseline did not result in significant improvements of lipids in participants who at baseline had normal blood levels of total cholesterol (<5.17 mmol/L).

Improvements in cardiometabolic risk factors appeared highest just after weight loss and up to one-year post-weight loss and progressively decreased over the subsequent years in the “Look AHEAD” study [30]. Some other studies from other populations have shown that the benefits derived from weight loss are not visible in the long term with or without larger percent weight losses occurring in the long term [43, 44]. In a ten-year study on the effect of lifestyle intervention on CVD outcomes [40], glycosylated haemoglobin concentrations improved most favourably during weight loss and in the first year after weight loss and regressed to baseline values. In line with this, it could be explained that although improvements in lipids and glucose profile of SWLM may have occurred just after the weight loss intervention, these may have reversed to baseline levels at the survey time which reflected a mean period of 6 months to 9 years after weight loss treatment (mean of which was 4.5 years). It is important to invest in studies that enhance the understanding of the long-term metabolism of lipids and glucose.

It has been demonstrated that higher losses usually ≥10% weight loss give the greatest improvement in cardiometabolic risk factors compared to smaller losses [19, 21]. Additionally, it has been observed that participants who have losses greater than 2% but lower than 5% also experience significant improvement in one cardiometabolic risk factor or the other [19, 21]. We therefore further categorised the percent weight loss achieved at follow-up in this study into as follows: ≤1.9% loss; 2.0–4.9% loss; 5–9.9% loss; ≥10% loss. Contrary to earlier findings by Brown and colleagues and Wing and colleagues, none of these categories showed a significant association with the blood parameters assessed (results not shown). This could be partly explained by the fact that these earlier studies examined the impact of short-term weight loss categories on lipid and glucose parameters, while we examined the impact of long term but similar weight loss categories on the same parameters. As mentioned earlier, the long-term effects of weight loss on cardiometabolic risk factors are usually not visible due to regression of these parameters to baseline levels [40, 43, 44].

In addition to the above explanations, the lack of significant difference in HDL readings for SWLM and UWLM could partly be attributed to the fact that a larger proportion of both SWLM and UWLM were either not involved in any form of physical activity or did not accumulate sufficient minutes of total activity per week. Augmented physical activity is known to effectively increase plasma HDL concentration [45–47]. Furthermore, HDL concentration was significantly increased when dieting was complimented by exercise compared to dieting alone [48]. Physical activity is known to stimulate the production of ATP binding cassette transporter A1 (ABCA1) [47] which helps with the transfer of phospholipids and cholesterol to apolipoprotein (apo) acceptors such as apo A-1 and apoE, resulting in the formation of pre-*β*HDL [49].

The lack of a significant difference observed in LDL concentration for SWLM and UWLM after controlling for lipid-lowering medication usage could partly be attributed to the type, dosage, and duration of intake of lipid-lowering medication and individual differences in medication response. In a study that reviewed the effectiveness of statins in lowering LDL, use of a higher dose (80 mg) of atorvastatin was more effective in lowering LDL cholesterol than using the regular dose of 40 mg atorvastatin or 40 mg of pravastatin [50]. Clinical studies typically evaluate the reduction in LDL cholesterol after administering statins for a period not less than 26 weeks and have recorded a significant improvement within this length of time [51]. Responses as low as 5% reduction in LDL concentration following usage of statins have been recorded. In some cases, some individuals have seen no improvement in LDL concentration due to resistance to statin treatment (familial hypercholesterolemia) [41].

By sheer ordering of mean concentrations of serum parameters assessed however, SWLM had more favourable values in LDL, HDL, FBG, and HbA1c compared to UWLM. It was also observed that, by sheer proportions, a greater percentage of SWLM compared to UWLM in our study had normal status for four major parameters (TC, TG, HbA1c, and LDL), thus reducing the burden of exposure to elevated levels of these parameters. We did not find studies that compared successful and unsuccessful weight loss maintainers on how they fared in terms of the proportions having normal values for these parameters. Brown and others however compared the CVD risk factor normalization status for short-term weight loss in the following categories: <5% weight loss; 5–10% weight loss; 10% weight loss in a sub-group of their participants who had elevated baseline values [21]. The prevalence of the normal FBG, TG, or HDL status in either SWLM or UWLM in our study was higher than that observed for each of the short-term weight loss categories assessed in the study by Brown and colleagues [21]. The lower prevalence reported for normal FBG, TG, and HDL by Brown and others compared to that of this study could be explained by the fact that their participants at baseline had higher values of these parameters, and although favourable changes occurred in mean values with reference to baseline values, it did not translate into normal values for a higher proportion of their participants. Brown and colleagues, however, had a greater proportion (50%–77.8%) of their high-risk group achieving normal LDL status in the different categories of weight loss assessed. In their study, the use of statins or other lipid-lowering medications was not accounted for, and therefore, it is possible that usage of these medications in the high-risk group may have contributed in part to the lowering of LDL values in their participants [21]. The “Look AHEAD” study investigated the effect of long-term weight loss (up to 4 years) on CVD risk factors in participants, given a lifestyle intervention of diet and physical activity [30]. In their study, 61% of participants achieved normal LDL values at four years. This was higher than that observed for SWLM (11.5%) in our study. Again, participants of the “Look AHEAD” study were on lipid-lowering medications, and this may have partly contributed to the higher proportions of participants having normal LDL status [30]. It was observed that only 13% of our study cohort used lipid-lowering medication, despite the high LDL values recorded at follow-up. This calls for increased education on the need to regularly check blood lipid status and take medications to lower cholesterol when values are high. Additionally, the low proportions of SWLM in our study having normal LDL status is of concern and suggests the need to further investigate the association between other factors such as dietary intake post-weight loss and LDL levels in successful weight loss maintainers for possible influences.

It is worth noting that a number of the studies that found a significant difference in CVD risk factors at weight maintenance had formal dietary and other lifestyle interventions continuing during the weight maintenance phase for all participants [30, 52–54]. In our study, no formal dietary intervention was given to participants after the weight loss programme except for those who chose to re-enrol at some stage after their initial “NWLP” weight loss bout, either for another bout of weight loss (26% of the cohort) or for weight loss maintenance (comprises 5.7% of the cohort). This intervention-free fallow period was necessary to ascertain how previous participants of the weight loss programme fared when intervention ends and also assess the importance they placed on their health post-intervention. A formal weight maintenance intervention however may improve compliance to the new diet and physical activity behaviours espoused during the weight loss programme and possibly make the impact of weight loss on lipid and glucose parameters more pronounced subsequently. The weight maintenance phase is typically characterised by recidivism in dietary and physical activity habits as revealed by the high prevalence of weight regain during this period [53, 55]. Wadden and colleagues [43] gave dietary and physical activity interventions for weight loss from day one till the forty-eighth week and thereafter left participants on their own with no weight loss maintenance or dietary intervention till week 100. Their findings in the sub-group with normal baseline total cholesterol (<5.17 mmol/L) were similar to that of this study where long-term maintenance of ≥5% weight loss did not show significant beneficial effects on blood parameter measurements at the maintenance phase.

The macronutrient composition of the diet has been shown to have some effect on lipid status. For instance, a low-fat diet (30% fat, 55% carbohydrate, and 15% protein) decreased LDL levels more favourably than a low-carbohydrate diet (20 g/day of carbohydrates with unlimited amounts of fat and protein), while the low carbohydrate diet decreased TG levels more favourably than the low-fat diet [56]. Under ad libitum conditions (unrestricted energy intake), a low-fat high-protein diet was found to increase glucose concentrations, while a low-fat high-carbohydrate diet increased triglyceride levels [57]. Lowering total fat intake and replacing saturated and trans fats with poly/mono-unsaturated fatty acids alongside decreasing cholesterol intake significantly improved the blood lipid profile in most individuals [58]. Foster and colleagues demonstrated that the effects of these macronutrients on blood lipid status was mostly short lived (3–6 months), pointing to the fact that blood lipid status partly reflects recent dietary intakes [56]. Although speculative, it is possible that both SWLM and UWLM were not as aggressive with dieting as they were during the weight loss phase and may have modified dietary macronutrient ratios at follow-up in ways that unfavourably regressed the improvements in lipid and glucose concentrations that may have been achieved by SWLM. Although 100% SWLM compared to 68.6% of UWLM practised the strategy of limiting food portions at meal times, this did not translate into a significantly greater proportion of SWLM having normal LDL levels. The other diet behaviours practised after the weight loss programme by majority of participants of this study were frequent consumption of high-fat/oily foods, frequent out-of-home eating, and consumption of less than five servings of fruits and vegetables per day. These are considered unhealthy dietary practises that could have contributed to the high proportions of participants (both SWLM and UWLM) having elevated LDL levels in this study. These findings further suggest that dietary fat restriction and the choice of healthier fats may be more important in reducing LDL and other lipid fractions in the body than just limiting food portions at meal times (includes limiting portions of atherogenic foods). The dietary behaviours reported in this study agrees with findings of the RODAM study, where urbanisation in Ghana had contributed to the westernisation of urban diets with a focus on high fats and highly processed foods [59]. Diets high in saturated fats increase blood levels of total and LDL cholesterol [60]. Foods prepared out of home are a source of high calories, sugar, salt, fats (high in saturated fatty acids) and are low in fruits and vegetables [61]. Adequate fruit and vegetable consumption can lower blood pressure via the supply of potassium and modulate cholesterol levels via the provision of flavonoids and other antioxidants, resulting in an overall decrease in the risk for CVDs [62]. The high prevalence of these unhealthy dietary behaviours among people who have previously lost weight calls for the urgent institutionalization of a weight loss maintenance support programme that includes healthy meal provision during the maintenance phase. This will enable weight losers get easy access to healthy meals low in fat and adequate in fruits and vegetables to partly contribute to the sustenance of the health benefits derived from weight loss. Although the choice of food remains the responsibility of the consumer, healthy food choices cannot be made if they are not available [61]. The enactment of nutrition policies and regulations for out-of-home foods will make these foods healthier, improve the food environment, and make it easier for the consumer to make healthier food choices.

TG concentrations observed for SWLM and UWLM were initially significantly different, however, after adjusting for the covariates earlier mentioned, and it was observed (through a linear regression analysis) that the differences were due to the influence of total percent weight loss at the end of weight loss programme, post-treatment time, gender, and follow-up BMI. Triglycerides have been identified as one of the lipids that is most sensitive to weight reduction [43], and this may explain why higher weight losses during the weight loss programme affected TG levels compared to the other lipid parameters assessed. A longer post-treatment time allowed triglycerides to rebound to baseline values in a systematic review investigating the impact of weight regain on cardiometabolic risk factors [39]. This supports findings of this study, where TG concentrations were significantly lower for SWLM who had a shorter post-treatment time compared to UWLM. Our study demonstrated that replacing of a male with a female favourably decreased triglyceride concentrations. This agrees with documentation in literature which points to the fact that women tend to have lower triglyceride concentrations due to accelerated production and enhanced clearance of triglycerides [63, 64]. In a study by Cugnetto and colleagues, BMI was observed to be a significant predictor of triglyceride concentration, and a unit increase in BMI was reported to increase triglyceride concentration by 0.02 units [65]. In another study, BMI was positively associated with triglyceride concentration [66]. These findings on BMI were consistent with that of this study, where every unit increase in BMI at the follow-up period resulted in 0.31 unit increase in TG concentration. Thus, despite the fact that SWLM were still obese at follow-up, they having a lower BMI at follow-up compared to UWLM partly contributed to the lower triglyceride concentration observed in SWLM.

The strength of this study resides in the fact that it reflects results of free-living populations who previously lost weight and is more typical of happenings in the real world compared to that of randomised controlled clinical studies. To the best of our knowledge, this is the first study comparing blood lipids and glucose concentrations of successful and unsuccessful weight loss maintainers in Ghana. Generalisation of this study to diabetics who at baseline may have a higher risk for CVD is unknown. The design of the study did not allow for blood parameter measurements at baseline and at the period marking the end of the weight loss intervention. This was a deficiency that did not allow for the assessment of within and intergroup (SWLM versus UWLM) short-term and long-term changes in blood parameter concentrations, except for follow-up intergroup comparison. This was necessary to lend a better understanding of the short-term and long-term metabolism of lipids and glucose. Although we looked at long-term impact, we used the average post-treatment time of 4.5 years and not specific time-course categories covering the range of post-treatment time observed in this study. A time-course assessment could have better revealed the association between specific time-course categories and the serum parameters measured. This was however outside the scope of the current study. Dietary intake at follow-up was not assessed. This would have made it possible to estimate macronutrient and energy intakes for successful and unsuccessful maintainers and link to findings for blood parameters assessed for possible associations. Dietary behaviours that were self-reported by participants as being practised were however assessed in the study.

## 5. Conclusions

This study showed that SWLM compared to UWLM had a significantly lower serum total cholesterol concentration. Additionally, LDL, HDL, FBG, and HbA1c concentrations were generally more favourable for SWLM compared to UWLM in terms of the sheer ordering of the group means. Furthermore, a greater proportion of SWLM had normal values for TC, TG, HbA1c, and LDL out of the six parameters measured. These outcomes, however, did not show a statistical significance. In spite of being successful at weight loss maintenance, a lower proportion of SWLM had normal LDL status compared to the proportions who had normal status for the other blood parameters studied. Weight loss maintenance success was associated with a decrease in triglyceride concentrations through metabolic processes connected with gender, post-treatment time, total percent weight loss achieved at the end of weight loss programme, and follow-up BMI status. Future studies must assess the impact of both short-term and long-term weight loss on serum lipids and glucose parameters which when elevated or low in the case of HDL can increase CVD risk. Studies investigating the association between post-weight loss dietary intake and serum lipids/glucose parameters, particularly LDL cholesterol, are warranted. Additionally, the differences in CVD morbidity and mortality for SWLM and UWLM are worth investigating prospectively.

## Figures and Tables

**Table 1:** Baseline anthropometric and demographic characteristics of successful and unsuccessful weight loss maintainers.

Variable	SWLM (*n*=26)	UWLM (*n*=86)	Significance
Age (mean ± SD)	42.4 ± 11.2	38.8 ± 8.2	0.036^¶^
Baseline weight (mean ± SD)	98.3 ± 12.4	94.2 ± 15.5	NS^¶^
Baseline height (mean ± SD)	1.64 ± 0.08	1.64 ± 0.06	NS^¶^
Baseline BMI (mean ± SD)	36.4 ± 4.6	35.1 ± 5.2	NS^¶^
Gender, *n* (%)			
Male	4 (15.4)	12 (14.0)	NS^ф^
Marital status, *n* (%)			
Not married	6 (23.1)	24 (27.9)	NS^ǂ^
Married	20 (76.9)	62 (72.1)	
Educational status, *n* (%)			
Below tertiary	1 (3.8)	10 (11.6)	NS^ф^
Tertiary and above	25 (96.2)	76 (88.4)	

SWLM = successful weight loss maintainers; UWLM = unsuccessful weight loss maintainers; NS = not significant. ^¶^Comparison of successful and unsuccessful weight loss maintainers based on ANCOVA; ^ǂ^comparison of successful and unsuccessful weight loss maintainers based on the chi-square test; ^Ф^comparison of successful and unsuccessful weight loss maintainers based on Fisher's exact test. Covariates controlled for in the ANCOVA model were total percent weight loss at the end of weight loss treatment and post-weight loss treatment time.

**Table 2:** Comparison of successful and unsuccessful weight loss maintainers on key weight variables, post-treatment time, and physical activity.

Variable	Mean ± SD (95% confidence interval)		Significance
	SWLM (*n*=26)	UWLM (*n*=86)	
Change in BMI from baseline to follow-up time (Kg/m^2^)^Ф^	−4.9 ± 2.4 [−5.9 to −3.9]	1.1 ± 1.9 [0.7 to 1.49]	<0.001
% weight loss at the end of weight loss programme^†^	−14.4 ± 6.9 [−17.2 to −11.6]	−5.5 ± 4.4 [−6.5 to −4.6]	<0.001
% weight loss achieved at follow-up time^ф^	−13.3 ± 6.2 [−15.8 to −10.8]	3.0 ± 5.0 [1.9 to 4.1]	<0.001
Post-treatment time (months)^¶^	45.2 ± 37.5 [37.9 to 55.2]	58.0 ± 31.1 [53.7 to 63.3]	NS
% weight regained median (IQR) (%)^Ѧ^	1.2 [−4.3 to 7.6]	9.1 [5.0 to 12.5]	<0.001
Minutes/week of total physical activity median (IQR) (minutes)^Ѧ^	130 [30 to 210]	100 [0 to 210]	NS

SWLM = successful weight loss maintainers; UWLM = unsuccessful weight loss maintainers; NS = not significant. Negative sign depicts a decrease in weight, BMI, or percent weight. ^Ф^Comparison of successful and unsuccessful maintainers based on ANCOVA with model adjusted for total percent weight loss at the end of weight loss treatment and post-weight loss treatment time; ^†^comparison of successful and unsuccessful maintainers based on ANCOVA with model adjusted for only post-treatment time; ^¶^comparison of successful and unsuccessful maintainers based on ANCOVA with model adjusted for only percent weight loss at the end of weight loss programme; ^Ѧ^comparison of successful and unsuccessful maintainers based on Mann–Whitney *Utest*.

**Table 3:** Comparison of successful and unsuccessful weight loss maintainers on fasting blood lipids, fasting blood glucose, and glycosylated haemoglobin concentrations.

Variable	Mean ± SD (95% confidence interval)		*P* value	
	SWLM (*n*=26)	UWLM (*n*=86)	Model 1	Model 2
Total cholesterol (mmol/L)	5.16 ± 0.99 [4.76–5.57]	5.59 ± 1.06 [5.37–5.82]	0.067	0.043^ƛ^^*∗*^
Low density lipoprotein (LDL) (mmol/L)	3.58 ± 0.92 [3.22–3.96]	3.87 ± 0.99 [3.66–4.08]	0.199	0.104^ƛ^
High density lipoprotein (HDL) (mmol/L)	1.22 ± 0.38 [1.07–1.37]	1.17 ± 0.32 [1.10–1.24]	0.512	0.365^ƛ^
Triglycerides (mmol/L)	0.79 ± 0.28 [0.68–0.91]	1.17 ± 0.51 [1.06–1.28]	<0.001^*∗*^	0.147^ƛ^
Fasting blood glucose (FBG) (mmol/L)	4.48 ± 0.72 [4.19–4.78]	4.73 ± 1.00 [4.52–4.95]	0.245	0.557^¶^
HbA1c (%)	5.52 ± 0.39 [5.36–5.68]	5.59 ± 0.59 [5.47–5.72]	0.555	0.411^¶^

SWLM = successful weight loss maintainers; UWLM = unsuccessful weight loss maintainers. Comparison of successful and unsuccessful maintainers is based on ANCOVA: Model 1, unadjusted model; Model 2, adjusted model. ^ƛ^Adjusted for total percent weight loss and post-treatment time and having hypercholesterolemia (categorical variable), cholesterol-lowering medication status (categorical variable), and total percent regain based on survey time, age, gender, and follow-up BMI; ^¶^adjusted for total percent weight loss and post-treatment time and having hypercholesterolemia (categorical variable) and total percent regain based on survey time, age, gender, and follow-up BMI; ^*∗*^significant *P* value.

**Table 4:** Standardised coefficient of covariates with regard to triglyceride concentration^†^.

Covariates	Standardised coefficients (beta)	*t*	*P* value
Percent weight loss at end of weight loss programme	0.192	2.013	0.047^*∗*^
Percent weight regained at survey time	0.004	0.034	0.973
Post-treatment time	0.202	2.195	0.031^*∗*^
Presence of hypercholesterolemia	0.082	0.947	0.346
Intake of cholesterol-lowering medication	0.049	0.550	0.584
Age	0.021	0.235	0.815
Gender	−0.270	−3.144	0.002^*∗*^
Follow-up BMI	0.307	3.083	0.003^*∗*^

^†^Based on a multiple linear regression model; ^*∗*^significant *P* value.

**Table 5:** Proportion of successful and unsuccessful weight loss maintainers achieving normal concentrations of serum lipids and glucose.

Variable	SWLM (*n*=26) % (*n*)	UWLM (*n*=86) % (*n*)	*P* value
Total cholesterol (<5.17 mmol/L)	53.8 (14)	36.0 (31)	0.105^†^
LDL (≤2.59 mmol/L)	11.5 (3)	8.1 (7)	0.695^¶^
HDL (≥1.03 mmol/L)	57.7 (11)	60.5 (34)	0.800^†^
Triglyceride (<1.7 mmol/L)	100.0 (26)	87.2 (75)	0.055^†^
FBG (3.6–6.4 mmol/L)	88.5 (23)	91.9 (79)	0.462^†^
HbA1c (<6.0%)	92.3 (24)	84.9 (73)	0.590^†^

SWLM = successful weight loss maintainers; UWLM = unsuccessful weight loss maintainers. ^†^Comparison of SWLM and UWLM based on Pearson's chi-square test; ^¶^comparison of SWLM and UWLM based on Fisher's exact test.

**Table 6:** The proportion of participants practising selected dietary behaviours.

Variable	Entire cohort (*n*=112) % (*n*)	SWLM (*n*=26) % (*n*)	UWLM (*n*=86) % (*n*)	*P* value
Not limiting fats/oils	76.8 (86)	73.1 (19)	77.9 (67)	0.609
Not limiting out-of-home eating	73.2 (82)	65.5 (17)	75.6 (65)	0.304
Not eating five servings of fruits and vegetables per day	54.5 (61)	16.4 (10)	83.6 (51)	0.062
Limiting food portion at meal time	75.9 (85)	100 (26)	68.6 (59)	0.001

SWLM = successful weight loss maintainers; UWLM = unsuccessful weight loss maintainers. Comparison of SWLM and UWLM is based on Pearson's chi-square test.

## Data Availability

Datasets analysed in the current study were used under Nutriline license and so are not publicly available. Data are, however, available from the corresponding author upon reasonable request and with permission of Nutriline. Requests for access to these data should be made to sayisi_addo@st.ug.edu.gh.
